# Total hip arthroplasty with tantalum rod extraction via direct anterior approach based on preoperative DCE-MRI and AI: a case report

**DOI:** 10.1097/MS9.0000000000003286

**Published:** 2025-05-21

**Authors:** Chen Zhiyuan, Sun Jiahao, Ma Bowen, Xia Tianwei, Shen Jirong

**Affiliations:** a Jiangsu Province Hospital of Chinese Medicine, Affiliated Hospital of Nanjing University of Chinese Medicine, Nanjing, China; b Nanjing University of Chinese Medicine, Nanjing, China

**Keywords:** AIHIP system, case report, DCE-MRI, direct forward approach, tantalum rod implantation

## Abstract

**Introduction and importance:**

Osteonecrosis of the femoral head (ONFH) is a debilitating condition characterized by compromised blood supply to the femoral head, leading to bone marrow or osteocyte death, and ultimately resulting in ischemia, necrosis, and potential collapse of the femoral head. Dynamic contrast-enhanced MRI (DCE-MRI) is a minimally invasive test that has the advantage of providing a microenvironmental picture of the femoral head that cannot be detected by conventional imaging (e.g., X-rays, CT, MR), and accurately assessing the viability and function of the local tissues, thus helping to select the appropriate surgical intervention. In this context, tantalum rod implantation has been employed as a treatment option for ONFH, aiming to provide structural support and potentially improve blood supply to the necrotic area. However, there are few reports in the literature on the evaluation of the efficacy of tantalum rod implantation in combination with DCE-MRI.

**Case presentation:**

A patient with bilateral SONFH, diagnosed according to the ARCO classification, presented with significant pain and limited mobility. Despite undergoing tantalum rod implantation in both hips, the patient reported minimal improvement in symptoms and continued to experience discomfort. Preoperative DCE-MRI was conducted to assess blood supply in the femoral heads, revealing inadequate perfusion in the necrotic areas. Given the poor response to tantalum rod implantation, a decision was made to proceed with DAA-THA to remove the tantalum rods and femoral heads. Intraoperative findings confirmed the presence of necrotic bone and lack of significant revascularization in the affected areas. The retrieved femoral head specimens were subjected to pathological and micro-CT analysis, which further confirmed the extent of necrosis and the inadequacy of blood supply.

**Clinical discussion:**

The use of tantalum rods in the treatment of ONFH is based on their osteoconductive properties and potential to promote revascularization. However, the success of this treatment is highly dependent on the preoperative blood supply status of the femoral head. DCE-MRI played a crucial role in this case by providing a clear picture of blood perfusion in the necrotic areas, which helped in identifying patients who may not benefit from tantalum rod implantation. The pathological and micro-CT analysis of the retrieved femoral head specimens provided additional insights into the reasons behind the inefficacy of tantalum rod implantation. Specifically, the lack of significant revascularization and the extent of necrosis highlighted the limitations of this treatment option in certain patient populations. The decision to proceed with DAA-THA was based on the patient’s poor response to tantalum rod implantation and the need for a definitive surgical intervention. The use of the AIHIP system for preoperative planning facilitated a precise and efficient surgical approach, minimizing trauma and optimizing postoperative recovery.

**Conclusion:**

This case study highlights the importance of comprehensive preoperative assessment using DCE-MRI in guiding the selection of surgical interventions for ONFH. Specifically, DCE-MRI can identify patients who may not respond favorably to tantalum rod implantation due to inadequate blood supply in the necrotic areas. In such cases, alternative treatment options such as DAA-THA should be considered.

## Introduction

Osteonecrosis of the femoral head (ONFH) is a joint disease with high morbidity and disability^[1,2]^, and its pathological progression is closely related to the blood supply within the femoral head^[^3^]^. 70% of patients will suffer from femoral head collapse within a few years, and THA can only be used eventually^[^4^]^. There are many early treatment measures for ONFH, such as core decompression^[^5^]^ and non-vascularized bone transplantation. Among them, tantalum rod implantation has been used clinically for more than ten years because of its minimally invasive and the advantages of combining with other surgical techniques^[^6^]^. However, with the wide application of tantalum rod, the patients’ condition did not improve after tantalum rod implantation and the cases requiring total hip replacement surgery increased^[^7,8^]^. DCE-MRI can quantitatively assess the microcirculatory function of the femoral head at an early stage^[^9^]^, and accurately show the perfusion, vascular permeability and capillary density of the femoral head, which can help to define the boundaries of the necrotic foci and the surrounding reaction zones. Although there are cases of hip preservation failure with tantalum rod implantation reported in the literature, there are few clinical reports focusing on the changes in the internal microenvironment of the femoral head^[^10^]^. In this paper, we report a middle-aged patient with bilateral femoral head necrosis who underwent bilateral hip tantalum rod implantation with poor postoperative outcome and eventually underwent DAA-THA. Preoperatively, we used DCE-MRI to assess microcirculatory changes in the femoral head and the AIHIP system for preoperative planning. We also performed pathology and micro-CT analysis of the femoral head specimens extracted during surgery to comprehensively analyze the reasons for the poor outcome of tantalum rod implantation, thus providing a reference for clinical practice.

**HIGHLIGHTS**

- This article uses DCE-MRI technology to evaluate the microcirculation changes of the femoral head after tantalum implant, which provides a new method for evaluating the effect of tantalum implant.
- Through the pathological and micro-CT analysis of the femoral head specimens removed during the operation, the possible reasons for the poor effect of tantalum rod implantation were discussed in depth, which provided a reference for clinical practice.

## Clinical data

### General information

**Figure 1.**
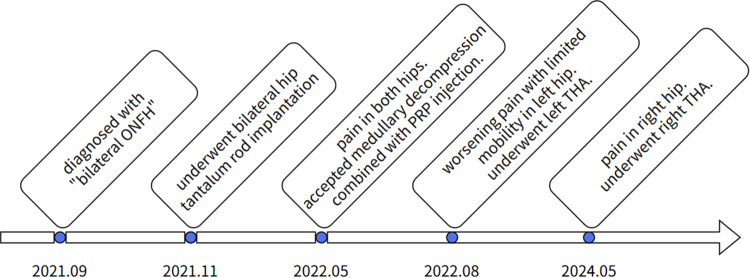
Timeline of the patient’s disease course.

**Figure 2.**
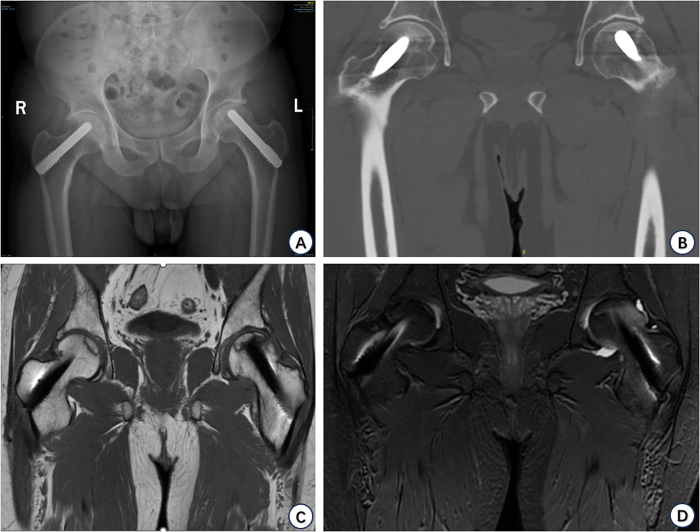
Patient’s preoperative imaging data. (A) Bilateral hip anteroposterior position (avascular necrosis of the femoral head bilaterally after tantalum rod implantation, secondary to osteoarthritis cystic degeneration of the hip joint; internal implants were visible). (B) CT coronal position (subchondral separation of the distal end of the tantalum rod on the left femoral head was visible, suggesting that instability of the femoral head mechanical structure was already present). (C and D) Coronal MRT1 and MRT2 phases (bilateral tantalum). (Bone growth and sclerotic bands are seen around the rods, and bone marrow edema is seen on the lateral side of the left femoral head).

**Figure 3.**
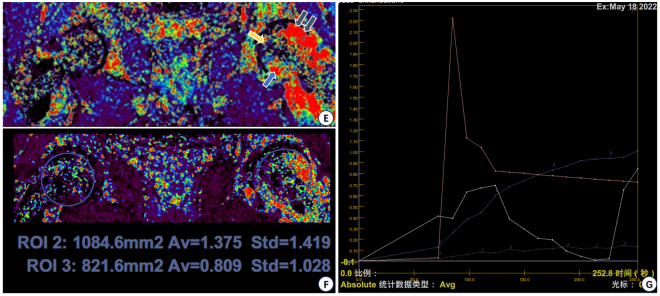
The patient’s preoperative DCE-MRI analysis results. (A) DCE-MRI Ve the parameter image (extensive fibrosis and vascularization around the necrotic area in the left femoral head: yellow arrow is the necrotic area, the part pointed out by the gray arrow corresponds to the bone marrow edema area, and the blue arrow suggests that there is an abnormal fibrosis in the lower part of the inner part of the tantalum rod). (B) DCE-MRI Ktrans the parameter image with quantitative analysis of the correlation (the quantitative analysis result of the left region of interest is much higher than that of the right side, suggesting that there is an abnormal inflammatory reaction in the left side of the head). (C) The in vivo concentration map of the contrast agent generated according to the calculation of the region of interest (the red line is the baseline, and it can be seen that the vascular permeability of the left side is significantly higher than that of the right side).

**Figure 4.**
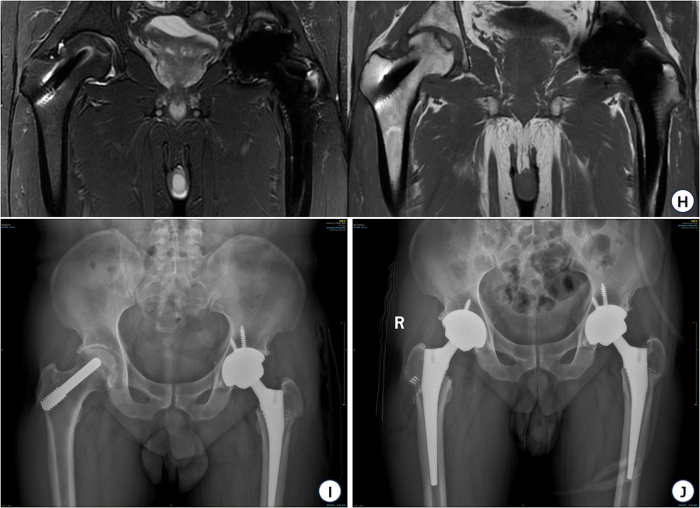
Postoperative imaging of the patient. (A) 2024-09, preoperative MRI of the right hip. (B) 2024-09, preoperative bilateral anteroposterior hip positions of the right hip. (C) 2024-09, postoperative bilateral anteroposterior hip positions of the right hip.

Patient, male, 47 years old, was diagnosed with “bilateral femoral head necrosis” in 2021-09 and underwent bilateral hip tantalum rod implantation in a local hospital in 2021-11. 2022-05, the patient was hospitalized in our department because of pain in both hips, and was treated with medullary decompression combined with PRP injection. 2022-08, the patient underwent left total hip replacement in our hospital because of worsening pain with limited mobility in the left hip. 2024-05, the patient underwent right total hip replacement in our hospital due to aggravation of right hip pain. The time line of the patient’s disease course, preoperative and postoperative imaging data are shown in Figs. 1–4.

### Surgical procedure

Based on the examination results of the patient, prepare to remove the tantalum rod and perform left hip replacement surgery. The preoperative surgical plan is carried out with the assistance of the AIHIP system.

**Figure 5.**
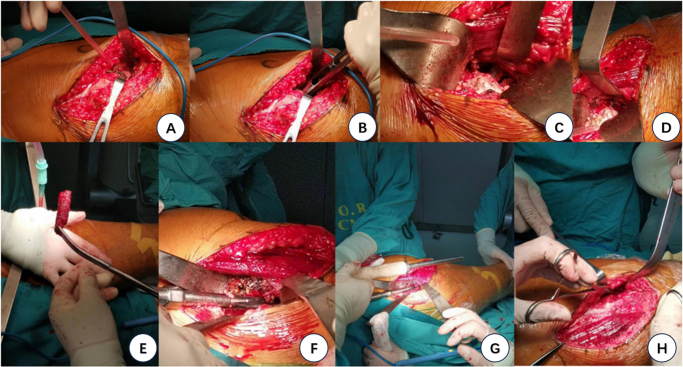
Surgical procedure and postoperative data. (A) The sarcolemma of vastus lateralis muscle was separated and cut through the direct anterior approach (DAA) incision to expose the end of the tantalum rod. (B) The part attached to the tantalum rod and the greater trochanter of femur was removed by a ring saw near the greater trochanter. (C) Osteotomy was performed on the base of the femoral neck from the front to the back (without cutting the tantalum rod to reduce debris), and the appropriate femoral calcar was retained according to the AI design (before breaking the tantalum rod with a bone knife). (D) After breaking the tantalum rod with a bone knife (taking the rod in sections). (E) The second half of the tantalum rod removed during the operation. (F) Reamer in the correct direction (the direction of the reamer held by the surgeon’s left hand). (G) Reamer should be used for reaming in the correct direction (the direction of the reamer held by the left hand of the surgeon), and should not mistakenly enter the bone tunnel left by the tantalum rod (the wrong reamer tunnel is the direction indicated by the locator held by the right hand of the surgeon), which will cause the reamer to penetrate the medullary cavity. (H) The sarcolemma of vastus lateralis was sutured first, then the sarcolemma of tensor fascia latae was sutured, and finally the subcutaneous and skin were sutured.

Cut open the subcutaneous tissue and fascia layer, explore and protect the lateral cutaneous nerve of the thigh, explore, separate and ligate the transverse branch of the lateral femoral artery, from the gap between the tensor fascia and rectus femoris muscle to the lower and distal ends of the anterior superior iliac spine. Cut off the tantalum rod connected to the large rotor with a circular saw. After exposing the joint capsule, make a circular incision and clean the synovium of the femoral neck to fully expose the femoral neck. Use a swinging saw to perform parallel osteotomy to remove the femoral head (including the proximal end of the tantalum rod), and then use Kocher forceps (i.e. toothed straight forceps, used for clamping thick tissue or clamping traction) and Rongeur forceps (i.e. bone biting forceps, used for biting and repairing bone tissue) to remove the distal end of the tantalum rod. When the acetabular bone expands to 49 mm, the acetabular bone wall oozes out uniformly. Insert and securely fix the test cup. Then insert the Pinnacle acetabular cup (diameter 50 millimeters) and insert two screws to enhance the stability of the acetabular cup. Open the femoral medullary cavity in the adduction and external rotation position and re-expand it. When the No. 10 handle expands again, it is firmly fixed after loading. After the short head test, the length of both lower limbs was consistent and the joint tension was qualified. Repeatedly clean the wound, suture layer by layer, and wrap it with sterile gauze. The surgery was very successful, and the unilateral surgery took 170 minutes (Fig. 5). The two operations were performed by the same surgeon.

After both surgeries, the patient’s condition was stabilized. After functional rehabilitation training, the Harris score recovered from 56.2 to 94.4 at the 1-year follow-up after the left hip operation, and the Harris score recovered from 73.5 to 90.5 at the 6-month follow-up after the right hip operation, which indicated that the hip joints were functioning well, and the review X-ray showed that the hip prostheses were in place and there was no sign of infection. The routine rehabilitation activities after surgery include: intermittent ankle pump exercises while resting in bed; On the first day after surgery, perform bedside standing and partial weight-bearing activities when tolerable; On the second day after surgery, gradually achieve complete weight-bearing using assistive devices; 3–5 days after surgery, practice walking with the help of a walking aid. Rehabilitation activities are conducted 2–3 times a day for 5–10 minutes.

### The femoral head specimens taken from the patients during the operation were analyzed

#### Micro-CT analysis

The main instrument is Hiscan XM Micro-CT (Suzhou Hisfeld Information Technology Co., Ltd.).

The femoral head sample, after removal, was scanned using the Quantum GX2 Micro-CT imaging system (PerkinElmer, USA) at a voltage of 50kV and a current of 160 μA, resulting in a voxel size of 50 μm.

#### Pathological analysis

Firstly, the gross pathological analysis of the femoral head removed during the operation was performed, and then the femoral head samples were decalcified to prepare tissue sections, which were stained by hematoxylin-eosin (HE) method for histological observation under the microscope.

**Figure 6.**
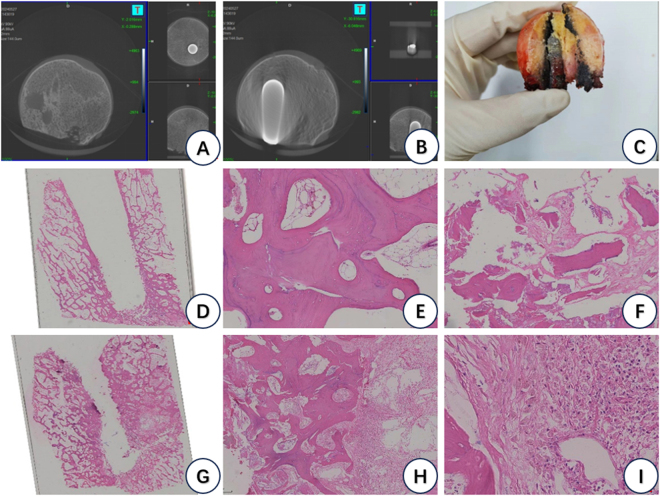
Analysis of femoral head specimens. (A and B): postoperative micro-CT; C: gross pathology of femoral head resected during surgery; (D to I): HE stained images. (D and G): sections of the coronal femoral head (×5 magnification); (E and H): tantalum rod sidewall section 1 (×40 magnification); (F and I): tantalum rod sidewall section 2 (×40 magnification).

Micro-CT results showed fractured trabeculae in the lateral femoral head, localized bone resorption outside the implantation area of the tantalum rod, and localized cystic degeneration; pathological analysis suggested that at 5x magnification, the trabeculae around the tantalum fibers seemed to be aggregated in patches (D, G), but there were no obvious osteoclasts or osteoblasts; when observed at 40x magnification, the aggregated trabeculae were likewise found, and some of the inflammatory cells (E, H); in some sections (F, I), we found a large number of macrophages. Interestingly, we seem to have failed to detect the presence of osteoblasts or osteoclasts (Fig. 6).

## Discussion

### Analysis of the causes of tantalum rod implantation failure

Tantalum rod implantation is considered an effective treatment for early avascular necrosis of the femoral head, offering stable mechanical support to the affected area, which can provide stable mechanical support for the necrotic area of the femoral head, effectively avoid the progress of ARCO stage II and III ONFH, and delay or even avoid THA^[^7,8,11^]^. Studies have shown that the smaller the necrotic area, the better the therapeutic effect of tantalum rod implantation^[^11^]^. Tanzer *et al*^[^12^]^ analyzed 15 cases of tantalum rod removed after operation failure and found that there was no new bone formation and blood vessel growth in a large area of femoral head necrosis, or the femoral head would most likely continue to collapse in the area where the tantalum rod was not supported, which may be one of the reasons for the failure of tantalum rod implantation.

We performed micro-CT examination and gross pathological examination of the removed femoral head during the operation, which showed fracture of the lateral trabecular bone, local bone resorption, local cystic degeneration, suspected large amount of adipose tissue filling around the distal end of the tantalum rod, and obvious adipogenic differentiation of bone marrow mesenchymal stem cells. In addition, HE staining showed that bone trabeculae gathered around the tantalum rod, no obvious osteoblasts or osteoclasts were observed, and obvious inflammatory cells appeared. Chronic inflammation has been shown to be a prominent feature of ONFH^[^13^]^. Tan *et al*^[^14^]^ found that an imbalance between M1 and M2 macrophages promotes the progression of nontraumatic ONFH; and necrotic bone products can induce upregulation of proinflammatory cytokines^[^15^]^. Mo *et al*^[^16^]^ found that the inflammatory response leads to tissue fibrosis and enhanced osteoclast activity, which subsequently leads to further structural damage to bone tissue. The above reports in the literature are consistent with the findings of the present study. Therefore, we hypothesize that tantalum rod implantation, as an invasive procedure, may activate a local inflammatory response in the short term, releasing pro-inflammatory factors, and the excessive release of these pro-inflammatory factors may exacerbate bone resorption. Tantalum rod implantation may also lead to a chronic inflammatory state, which may activate osteoclasts and lead to bone destruction, accelerating the progression of ONFH and ultimately leading to failure of hip preservation surgery.

Postoperative examination likewise revealed a suspected large amount of adipose tissue filling around the distal end of the tantalum rod, and significant adipogenic differentiation of bone marrow mesenchymal stem cells (BMSCs). Kong *et al*^[^17^]^ noted that elevated adipogenic differentiation of BMSCs is closely associated with non-traumatic osteonecrosis of the femoral head, and that glucocorticoids can disrupt the normal differentiation of BMSCs and accelerate adipogenesis^[^18^]^. There is some kind of balance between adipogenesis and osteogenesis^[^19^]^, and the mechanism may be potentially relevant for the treatment of ONFH. These results are consistent with our findings. The porous structure of tantalum rods may promote osteoblast differentiation, provide mechanical support for necrotic areas, and reduce intra-femoral head pressure, thus improving local microcirculation. However, the patient’s preoperative MR showed bone marrow edema within the patient’s head, suggesting that the pressure-reducing and differentiation-promoting effects of tantalum rods did not take effect. Previous studies have shown that bone marrow adipocyte proliferation is closely related to local ischemia and elevated pressure. The failure of tantalum rod implantation to effectively improve local intramedullary hypertension and promote osteogenic differentiation ultimately led to the emergence of a large amount of adipose tissue at the distal end of the tantalum rod, further weakening the mechanical support of the necrotic area, which may be one of the reasons for the failure of this tantalum implantation.

Although the pathogenesis of non-traumatic osteonecrosis of the femoral head is still unclear, changes in intramedullary circulation are known to be involved, and stenosis or occlusion of the vessels in the femoral head is the direct cause of non-traumatic osteonecrosis of the femoral head^[^20^]^. DCE-MRI is a minimally invasive technique to assess the blood perfusion status of lesions^[^21^]^. This technique mainly reflects the distribution of microvessels and capillary blood perfusion, which is used to evaluate the vitality and function of local tissues, and can specifically evaluate the perfusion status of tissues and organs^[^22^]^. Combined with this patient’s preoperative CT, DCE-MRI localization map and pseudo-color map, it was found that although the tantalum rod was implanted below the necrotic area, the mechanical environment and microcirculatory perfusion within the head were not effectively improved. The patient’s left femoral head was hyperperfused, with abnormal blood flow, increased vascular permeability, and further deterioration of the intra-head microenvironment, which successively led to the patient’s severe pain in the affected hip, which may have been the main reason for the failure of tantalum implantation in this patient^[^23^]^. We believe that DCE-MRI can adequately assess the patient’s preoperative condition by quantifying the femoral head microcirculation parameters, clarifying the blood supply status of the necrotic region, and identifying underperfusion of the necrotic region or abnormal hyperperfusion around the necrotic region. If the patient in this case had received DCE-MRI prior to hip-sparing surgery, the outcome might have been very different.

These results suggested that tantalum rod implantation did not fundamentally cure the patient’s femoral head necrosis, but could only provide mechanical support, and the patient’s femoral head necrosis was still developing, and eventually the pain became worse, and DAA-THA was needed. This result reflects the limitation of tantalum rod implantation.

### Points for attention in DAA-THA after tantalum rod implantation

The removal of tantalum rod is the main problem that needs to be dealt with during THA for patients with femoral head necrosis after tantalum rod implantation. Firstly, it is difficult to remove the implanted tantalum rod due to its lack of tissue adhesion and low degradation rate. Secondly, during THA, the amount of intraoperative blood loss in patients with tantalum rod implantation was significantly higher than that in patients without tantalum rod implantation. In addition, the long-term wear and corrosion of tantalum rod in the body will produce a small amount of metal ion accumulation, which will increase the risk of toxic reactions in patients.

Zhao *et al* ^[^24^]^ compared anterior and posterior approaches and found that orthotopic surgery was a simple and effective method of removing trabecular metal rods using a bone drill. In this case, DAA access was used to remove the tantalum rods. We believe that the tantalum rods can be easily removed by using the following techniques. (1) Remove the tantalum rod correctly. During the operation, the tantalum rod was not directly removed, but the tail end of the tantalum rod was removed with a special ring saw, and then the base of the femoral neck was cut with a pendulum saw. Kocher forceps and Rongeur forceps were used to remove the tantalum rod in sections, which shortened the operation time and reduced tissue damage. (2) Attention should be paid to avoid misjudgment during pulp opening, which may lead to the wrong insertion of the reamer. The reamer should be reamed in the correct direction (the direction of the reamer held by the left hand of the surgeon), and should not mistakenly enter the bone tunnel left by the tantalum rod (the wrong reamer tunnel is the direction indicated by the locator held by the right hand of the surgeon), which may cause the reamer to penetrate the medullary cavity. (3) Finally fill the bone tunnel left when the tantalum rod was implanted.

### Potential for integration and application of DCE-MRI with AIHIP

The synergy between DCE-MRI and AIHIP has a broad prospect in the field of orthopedics. In terms of preoperative assessment and individualized treatment, DCE-MRI can effectively screen high-risk patients by quantifying the microcirculation parameters of the femoral head, providing a key basis for diagnosis, intervention and prognosis; AIHIP system can generate a 3D model based on the CT data, and automatically optimize the implantation position and angle of the prosthesis by combining with the patient’s anatomical structure, bone mineral density, and necrosis range. The combination of the two is expected to avoid the limitations of traditional imaging, which is “morphology-based,” and realize the accurate assessment of “function + structure” in two dimensions, which significantly reduces the failure rate of surgery. Regular postoperative DCE-MRI examination can assess the recovery of blood supply of the femoral head, and early detection of bone resorption, loosening of the prosthesis and other complications.

Digital orthopedic technology is being more and more applied to the clinical practice of joint surgery. In the author’s opinion, in the future, DCE-MRI and AI system can work together to establish an integrated standard process of “image acquisition – data analysis – surgical planning – postoperative management,” and through deep learning and continuous optimization of algorithms, it can integrate postoperative images, functional scores (e.g., Harris scores), and biomechanical data to build prognostic prediction models, identify patients at high risk of recurrence, and identify patients at high risk of recurrence. It can identify patients at high risk of recurrence and recommend personalized rehabilitation programs. Although the application of DCE-MRI still needs to overcome the challenges of technology integration, data sharing and clinical validation, it is an important direction for the future development of joint surgery, and the synergy between DCE-MRI and AIHIP has a bright future in orthopedics. In terms of preoperative assessment and individualized treatment, DCE-MRI can effectively screen high-risk patients by quantifying the microcirculation parameters of the femoral head, which provides a key basis for diagnosis, intervention treatment, and prognosis; the AIHIP system can generate a three-dimensional model based on the CT data, and combine with the patient’s anatomical structure, bone mineral density, and the extent of necrosis, to optimize the prosthesis implantation position and angle automatically. The combination of the two is expected to avoid the limitations of traditional imaging, which is “morphology-based,” and realize the accurate assessment of “function + structure” in two dimensions, which significantly reduces the failure rate of surgery. Regular postoperative DCE-MRI examination can assess the recovery of blood supply of the femoral head, and early detection of bone resorption, loosening of the prosthesis and other complications.

Digital orthopedic technology is being more and more applied to the clinical practice of joint surgery. In the author’s opinion, in the future, DCE-MRI and AI system can work together to establish an integrated standard process of “image acquisition – data analysis – surgical planning – postoperative management,” and through deep learning and continuous optimization of algorithms, it can integrate postoperative images, functional scores (e.g., Harris scores), and biomechanical data to build prognostic prediction models, identify patients at high risk of recurrence, and identify patients at high risk of recurrence. It can identify patients at high risk of recurrence and recommend personalized rehabilitation programs. Although its application still needs to overcome the challenges of technology integration, data sharing, and clinical validation, it is an important direction for the future development of joint surgery.

## Conclusions

In the above article, we synthesized DCE-MRI, micro CT and pathological analysis to analyze the possible causes of tantalum rod implantation failure from multiple perspectives, combined with AIHIP for preoperative design, and ultimately successfully performed hip replacement from the DAA approach. Despite the extremely small sample size of the clinical cases reported here, the ability of DCE-MRI in assessing microcirculatory changes in the femoral head of patients is encouraging. Hip preservation is an important topic in the treatment of femoral head necrosis, and how to fully utilize the roles of DCE-MRI and AI is a question we should address next.

## Data Availability

The relevant research data generated for this manuscript are not deposited in public databases. The datasets used during the current study are available from the corresponding author on reasonable request.
